# Gender and regional disparities of tuberculosis in Hunan, China

**DOI:** 10.1186/1475-9276-13-32

**Published:** 2014-04-27

**Authors:** Mengshi Chen, Abuaku Benjamin Kwaku, Youfang Chen, Xin Huang, Hongzhuan Tan, Shi Wu Wen

**Affiliations:** 1 Department of Epidemiology and Health Statistics, School of Public Health, Central South University, Changsha, Hunan 410008, P. R. China; 2 Hunan Institute of Tuberculosis Prevention and Treatment, Changsha, Hunan 410012, P. R. China; 3 Department of Obstetrics & Gynecology and Department of Epidemiology & Community Medicine, University of Ottawa, The Ottawa Hospital 501 Smyth Road, Ottawa, Ontario, Canada

**Keywords:** Health Disparity, TB, Gender, Region

## Abstract

**Introduction:**

Major efforts have been made to improve the health care system in Hunan province, China. The aims of this study were to assess whether and to what extent these efforts have impacted on gender and regional disparities of Tuberculosis (TB) incidence in recent years, especially for less developed areas.

**Methods:**

We obtained data from the 2005–2009 China Information System for Disease Control and Prevention (CISDCP)to conduct this study in Hunan province. Counties within the province were divided into four regions according to quartiles based on the 2007 per capita GDP. Index of Disparity (ID) and Relative Index of Inequality (RII) were used to measure the disparities of TB incidence in relation to gender and region. Bootstrap technique was used to increase the precision.

**Results:**

The average annual incidence of TB was 111.75 per 100,000 in males and 43.44 per 100 000 in females in Hunan. The gender disparity was stable, with ID from 42.34 in 2005 to 43.92 in 2009. For regional disparity, ID, RII (mean) and RII (ratio) decreased significantly from 2005 to 2009 in males (P < 0.05) but remained stable among the female population.

**Conclusions:**

As interventions such as introduction of the New Rural Cooperative Scheme put in place to reduce health disparities in China, regional disparity in relation to incidence of TB decreased significantly, but the gender disparity remains in the Hunan province.

## Introduction

In recent years, health disparity in different subgroups has become a major public health problem in the world [1-4]. In 2001 the Netherlands National Center for Health Statistics issued a monograph with 11 guidelines for reporting health disparities [5]. Keppel and colleagues define health disparity as "difference in incidence, prevalence, mortality, and burden of disease and other adverse health conditions that exist among specific population groups" [6].

Tuberculosis has been associated with low levels of education, poverty and income inequality [7,8]. Higher TB incidence has also been reported among males compared with females [9]. The 2009 male to female ratio (MFR) of notified new sputum smear positive TB cases in the different World Health Organization (WHO) regions were 1.35: 1.00 in Africa; 1.49: 1.00 in the Americas; 2.03: 1.00 in South-East Asia; 2.16: 1.00 in Europe; and 2.40:1.00 in China [9]. Disparity of TB incidence in the different WHO regions has also been reported [9]. It is estimated that 90% of global TB cases and deaths occur in developing countries, and the incidence of TB in developed countries is far lower than that in developing countries [10]. The China National Random Survey in 2010 showed that the prevalence of TB varied among different gender groups and regions. The prevalence in males was higher than in females whilst prevalence in eastern China, which is more developed, was lower than in western China, which is less developed [11]. These studies adopted MFR to describe gender disparity, and discovered regional disparity in prevalence among different socioeconomic subgroups.

Hunan, a province located in central south of China with a population size of 64 million in 2009, is a developing province in China. In 2002, only 2.76% of the province’s financial budget was spent on health [12]. Also, 96% of rural residents lacked medical insurance resulting in higher TB prevalence in 2003. Following these challenges, a number of measures were put in place to reduce the TB epidemic and disparities. In addition to the Directly Observed Treatment Short-course (DOTS) strategy implemented in the province in 1992, and reaching a coverage rate of 95% at the end of 2001 [13], a New rural Cooperative Medical Scheme (NCMS) was established in 2003 to improve the medical situation of rural residents. The coverage rate for NCMS, which is a public health insurance scheme, increased from 17.96% to 99.10% between 2005 and 2009 whilst funds for the scheme increased from 171 204 600 *yuan* (about $ 26 750 000) to 4 784 828 200 *yuan* (about $ 747 630 000) [14]. The number of rural doctors per thousand rural population also increased from 0.67 in 2003 to 0.84 in 2009 [15]. Did all the aforementioned efforts help reduce gender and regional disparities in TB, in Hunan province of China?

We attempted to fill this information gap by describing disparities of TB incidence in relation to gender and region as well as trends of disparity from 2005 to 2009 in the province using disparity indicators.

## Materials and methods

We obtained Hunan TB data from the 2005–2009 China Information System for Disease Control and Prevention (CISDCP). The CISDCP system covers the whole population and all legal reporting infectious diseases, including TB. Case detection follows WHO recommended passive case-finding guidelines. Individuals with TB related symptoms should be identified when they seek care at a general health facility, and should be referred to the specialized TB dispensary for diagnosis, treatment and case management. All the TB patients were diagnosed according to a set of diagnostic criteria [16]. According to the law of prevention and control of infectious diseases, physicians are obliged to report every newly diagnosed TB patient to the local center for disease prevention and control (CDC). The local CDC is responsible for uploading data onto the CISDCP system. The data concerning population and economic indicators (including population size, Gross domestic product (GDP), etc.) in this study were extracted from "*Hunan Statistical Yearbook 2010*" [17]. Counties in the province were classified into four regions according to quartiles based on the 2007 per capita GDP [18].

Index of Disparity (ID) and Relative Index of Inequality (RII), which were recommended by the National Institutes of Health Strategic Research Plan to Reduce and Ultimately Eliminate Health Disparities, were used as disparity indicators in this study [6]. These indicators were commonly used for gender and regional disparity measurement.

**ID**, which is suitable for categorical and ordinal variables, summarizes the average difference between several group rates, and expresses the summed differences as a proportion of the reference rate. If there is no disparity, ID equal 0. This index is calculated as *ID* = ∑ {|*y*_*j*_ - *y*_*ref*_| ÷ *N*} ÷ *y*_*ref*_ × 100 where y_*j*_ indicates the measure of health status in the *jth* group, and y_*ref*_ is the health status indicator in the reference population. Theoretically, any group can be chosen as the reference group. In this study, we selected the total population rate as the reference group [6].

**RII,** which is suitable for ordinal variable only, is a regression-based measure. Regression-based approaches are used to plot an ordinal regional socioeconomic status measure from the smallest regional socioeconomic status category to the largest, while incorporating appropriate population weights for each category. In this study regional socioeconomic status groups were first ranked from lowest to highest on the horizontal axis, and the cumulative proportion of the population for each group was computed. The size of each group was proportional to its population size on a scale from 0 to 1. Then, the prevalence (y) for each regional socioeconomic status group was plotted at the midpoint of each regional socioeconomic status group’s proportion range. Weighted least squares were used with the weights proportional to the population size of each group. The fitted regression line had the form *y* = a + b*x*. RII can be formed in one of two ways, denoted as RII(mean) and RII(ratio). Two versions of the RII are all related to the linear regression coefficient b. *RII*_(*mean*)_ = *b* ÷ *u*, where u is the average health status of the population, and *RII*_(*ratio*)_ = *a*/(*a* + *b*), which is the value of y at the intercept (x = 0) divided by the value of y at x = 1 [6,19].

We used a resampling or bootstrap technique to increase the precision of measure [6]. For each year, gender, and region, we used the observed rate and its standard error to re-estimate their rate 100 times under the condition of random normal distribution. Based on these group rates, the same number of summary measure estimates was generated, and the distribution of these estimates was used to compute the standard error of the summary measure. The u test was used to compare TB incidence in different gender population subgroups. Linear regression was used to examine the significance of disparity indicator trends over the years [20]. SAS software, version 9.2, was used for data analysis.

## Results

The average annual incidence of TB was 78.88 per 100,000 population in Hunan province during 2005–2009, with 111.75 per 100,000 population for males, and 43.44 per 100,000 populations for females (Table 1).

**Table 1:** TB incidence in Hunan, 2005-2009

	**Male**			**Female**			**Total**			**Incidence ratio**
	**Population(×1000)**	**Cases**	**Incidence (1/100000)**	**Population(×1000)**	**Cases**	**Incidence (1/100000)**	**Population(×1000)**	**cases**	**Incidence (1/100000)**	**(male: female)**
2005	34 707.5	37 239	107.29^#^	32 269.5	13 691	42.43	66 977	50 930	76.04	2.53
2006	34 905.9	39 492	113.14^#^	32 415.1	14 945	46.11	67 321	54 437	80.86	2.45
2007	35 133.5	39 566	112.62^#^	32 547.5	13 885	42.66	67 681	53 451	78.97	2.64
2008	35 338.7	39 745	112.47^#^	32 718.3	14 082	43.04	68 057	53 827	79.09	2.61
2009	35 493	40 173	113.19^#^	32 959.0	14 167	42.98	68 452	54 340	79.38	2.63
Average	35 115.7	39 243	111.75^#^	32 581.9	14 154	43.44	67 697.6	53 397	78.88	2.57

^#^compared with female, P < 0.05.

The result of linear regression showed that gender disparity in TB incidence was stable: ID = 42.34 in 2005 and 43.92 in 2009. (*P* = 0.171, Table 2).

**Table 2:** ID of gender disparity of TB incidence in Hunan, 2005-2009

	**ID**	
	**Mean**	**Standard error**
2005	42.34	3.26
2006	41.15	3.18
2007	44.11	3.47
2008	43.85	3.32
2009	43.92	3.31

The time trend was tested by linear regression, P = 0.171.

The average annual incidence of TB was 88.47 per 100, 000 population; 79.94 per 100,000 population; 75.05 per 100,000 population; and 68.64 per 100,000 population in low, middle, high, and upper high per capita GDP regions, respectively (Table 3).

**Table 3:** TB incidence among different SES regions in Hunan, 2005-2009

**Regional SES**		**2005**	**2006**	**2007**	**2008**	**2009**	**Mean**
Low	Population(×1000)	16 978.67	17 059.14	17 123.29	17 211.62	17 352.58	17 145.06
	cases	14 685	15 558	15 275	15 370	14 951	15 168
	incidence (1/100,000)	86.49	91.2	89.21	89.3	86.16	88.47
Middle	population(×1000)	21 030.78	21 118.6	21 238.3	21 342.68	21 473.39	21 240.75
	cases	16 067	17 155	16 965	17 125	17 582	16 979
	incidence (1/100,000)	76.4	81.23	79.88	80.24	81.88	79.94
High	population(×1000)	17 480.99	17 543.85	17 644.44	17 735.65	17 824.9	17 645.97
	cases	12 656	13 691	13 067	13 109	13 693	13 243
	incidence (1/100,000)	72.4	78.04	74.06	73.91	76.82	75.05
Upper high	population(×1000)	11 486.56	11 599.41	11 674.97	11 767.06	11 801.12	11 665.82
	cases	7 522	8 034	8 145	8 223	8 115	8 008
	incidence (1/100,000)	65.49^*^	69.26^*^	69.76^*^	69.88^*^	68.76^*^	68.64*

^*^time trend, P < 0.05.

**Figure 1:**
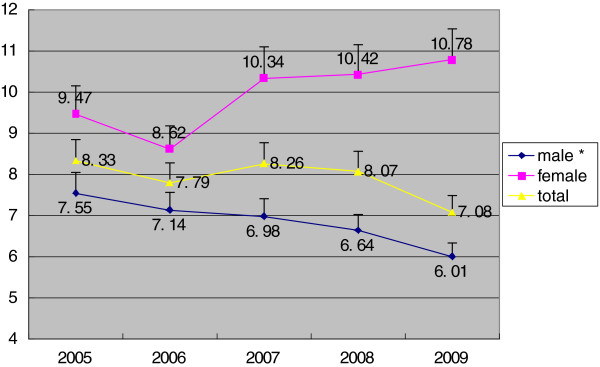
**ID of TB incidence in different SES regions in Hunan, Stratified by gender, 2005–2009.** *time trend, P<0.05.

**Figure 2:**
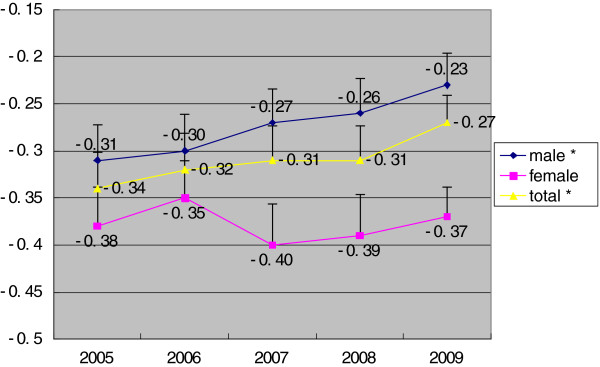
**RII (mean) of TB incidence in different SES regions in Hunan, Stratified by gender, 2005–2009.** *time trend, P<0.05.

**Figure 3:**
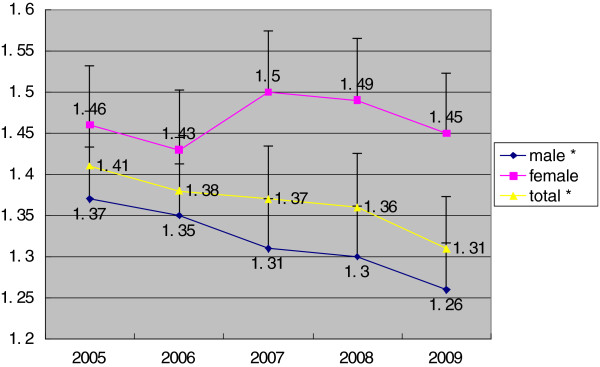
**RII (ratio) of TB incidence in different SES regions in Hunan, Stratified by gender, 2005–2009.** *time trend, P<0.05.

The result of linear regression analysis showed that disparity of TB incidence in different SES regions decreased significantly among the male population: from ID = 7.55 in 2005 to ID = 6.01 in 2009 (P < 0.05), but remained stable among the female population as well as the entire population between 2005 and 2009(P > 0.05) (Figure 1). The RII (mean) and RII (ratio) of regional disparity decreased among the male population as well as the entire population from 2005 to 2009 (P < 0.05), but was stable in female population (P > 0.05) (Figures 2 and 3).

## Discussion

Our study is the first to report on gender and regional disparity as well as time trends in relation to TB incidence in the Hunan province of China. Analysis of the 2005 – 2009 surveillance data from the China Information System for Disease Control and Prevention showed that the average annual incidence of TB was 111.75 per 100,000 in males and 43.44 per 100 000 in females, yielding a rate ratio of 2.57 in males compared to females. Gender disparity in TB incidence was stable during the years of study. Although TB incidence decreased significantly with increasing regional socioeconomic status (p < 0.05), regional disparities in incidence reduced among males and the entire population but not among females. The observed higher TB incidence in males compares well with WHO reports [9].

Gender disparity in TB incidence may be caused by differences in burden of cigarette smoking, which is a predisposing factor for TB. In China, the rate of cigarette smoking was about 66.0% in men but only 3.08% in women [21]. Risk of TB infection has also been associated with the increasingly differentiated social roles for adolescents as they grow up into adulthood. Adolescent males, who are generally risk takers, are at higher risk of TB infection [22]. Some reports have also shown that estradiol (a female hormone) could enhance immunity, while testosterone (a male hormone) inhibits immunity [23,24]. Trend analysis showed no reduction in gender disparity of TB incidence from 2005 to 2009. This suggests that current TB control policy, which focuses on improving detection and cure rates, has limited effect on gender inequality in TB incidence, and therefore needs to be revised.

The significant decline of TB incidence with increasing regional socioeconomic status (P < 0.05), compares well with findings of the China National Random Survey in 2010 [11]. Low income has been associated with poor living conditions; poor nutritional status; and inadequate access to health care services leading to increased susceptibility to mycobacterium TB [25-27]. Furthermore, under-reporting of TB was generally higher in low SES populations compared to high SES populations during the 2010 national survey [11]. This suggests that the regional disparity in TB incidence observed in our study may be wider.

Regional disparity in TB incidence declined among males from 2005 to 2009, this suggests that current DOTS strategy and NCMS program is effective in declining regional disparity in TB incidence. But it is not effective in females. The NCMS program has some effects in reducing income-related health inequity [28]. But it's still hard for women to get health care, especially for old women [29]. For example, in rural Hunan, 36% of women preferred Buddha rather than medical treatment after getting TB [30]. We should take time and effort to improve access to health care among females, such as health education.

Our study is limited in the fact that data used came from a surveillance system, which did not allow for a direct measurement of under-reporting of TB. The National Random Survey in 2010 provided some reports on under-reporting, however, the under-reporting do not negate our findings. For instance, the survey reported that under-reporting of TB was higher in males than females. Our finding of higher TB incidence among males can therefore be said to be real.

## Conclusions

In conclusion, our study has shown that current DOTS strategy and NCMS program is effective in declining regional disparity in TB incidence; but the gender disparity in relation to TB incidence in the Hunan province remains. This finding indicated that we must pay more attention to females in developing policies/interventions for the reduction of gender disparities in TB incidence, not just rely on DOTs and NCMS program. Hunan province is a representative of developing regions, thus our finding may be referential for other developing regions or countries.

## Competing interests

The authors declare they have no competing interests.

## Authors’ contributions

MC and HT designed the study and drafted the manuscript. MC, ABK and XH carried out the data analysis. YC and SWW supervised data analyses and results reporting. SWW assisted in the development of the research question and revision of the article. All authors read and approved the final manuscript.
